# Supplementary material for: Mechanisms governing the pioneering and redistribution capabilities of the non-classical pioneer PU.1
Source: Nat Commun. 2020 Jan 21;11:402. doi: 10.1038/s41467-019-13960-2 (PMC6972792; doi:10.1038/s41467-019-13960-2)
Supplement: Supplementary file 7 — Source data [file 41467_2019_13960_MOESM7_ESM.zip › Source_Data/FigureS2/FigureS2B_MotifScanOutput/KmeansCluster7/homerResults/motif56.similar.html]

motif56

## Information for motif56

A
C
G
T
A
G
T
C
A
T
G
C
A
C
G
T
A
T
G
C
A
T
G
C
A
C
G
T
A
G
T
C
  
Reverse Opposite:  

A
C
T
G
C
G
T
A
A
T
C
G
A
T
C
G
C
G
T
A
A
T
C
G
A
C
T
G
C
G
T
A
  

|  |  |
| --- | --- |
| p-value: | 1e-20 |
| log p-value: | -4.742e+01 |
| Information Content per bp: | 1.898 |
| Number of Target Sequences with motif | 319.0 |
| Percentage of Target Sequences with motif | 9.94% |
| Number of Background Sequences with motif | 2424.4 |
| Percentage of Background Sequences with motif | 5.55% |
| Average Position of motif in Targets | 104.2 +/- 53.1bp |
| Average Position of motif in Background | 102.7 +/- 82.6bp |
| Strand Bias (log2 ratio + to - strand density) | -0.1 |
| Multiplicity (# of sites on avg that occur together) | 1.12 |
| Motif File: | file (matrix) reverse opposite |

### Similar de novo motifs found

|  |  |  |  |  |  |  |  |
| --- | --- | --- | --- | --- | --- | --- | --- |
| Rank | Match Score | Redundant Motif | P-value | log P-value | % of Targets | % of Background | Motif file |
| 1 | 0.768 | C T A G C G T A T C A G C T A G C G T A C T A G T C A G C G T A A T C G T C A G C T G A A T C G | 1e-15 | -36.163039 | 3.61% | 1.46% | motif file (matrix) |
